# Critical Care Transthoracic Echocardiography: Atrial Sinus Rhythm During Ventricular Fibrillation

**DOI:** 10.1177/2324709614524945

**Published:** 2014-02-19

**Authors:** Peter Mark Schulman, Jill M. Gelow, Matthias J. Merkel, James O. Mudd, Kevin Wei, Michael P. Hutchens

**Affiliations:** 1 Oregon Health & Science University, Portland, OR, USA

**Keywords:** LVAD, TTE, intensivist, ventricular fibrillation, sinus rhythm

## Abstract

A 70-year-old man with end-stage heart failure and a continuous-flow left ventricular assist device received repeated electrical discharges from his biventricular implantable cardiac defibrillator. Although the electrocardiogram demonstrated ventricular fibrillation, the patient was awake and in no distress. A focused transthoracic echocardiogram was performed to assess ventricular function revealing simultaneous atrial sinus rhythm and ventricular fibrillation. This clinical scenario highlights a unique clinical finding, the complexity of the care of continuous-flow left ventricular assist device patients, and the challenges of intensivist-performed bedside focused transthoracic echocardiogram.

## Case Presentation

A 70-year-old man with end-stage heart failure and a continuous-flow left ventricular assist device (LVAD) received repeated electrical discharges from his biventricular implantable cardiac defibrillator (BiV-ICD) while working in his office. His past medical history included atrial fibrillation requiring AV node ablation and ventricular tachycardia with a history of high defibrillation thresholds requiring placement of an additional defibrillator coil in his azygous vein. His antiarrhythmic therapy included sotalol and carvedilol. He presented to a referring emergency department where he reported chest discomfort but denied other complaints. Electrocardiogram demonstrated ventricular fibrillation (VF). The patient was transferred to our hospital.

**Figure 1.**
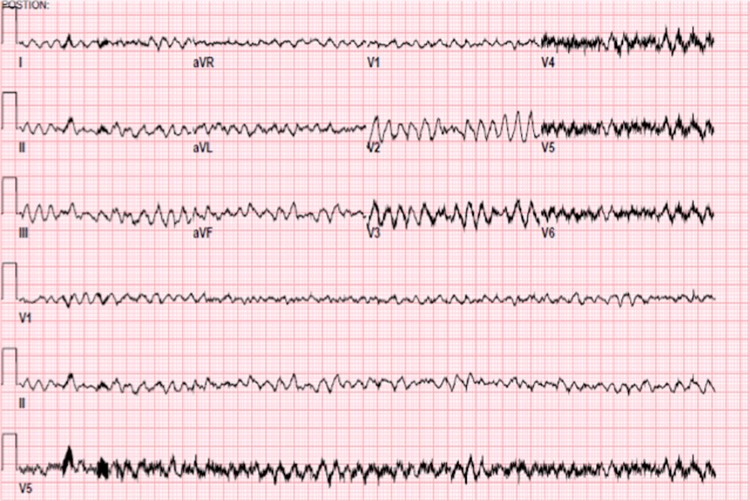
Electrocardiogram demonstrating ventricular fibrillation.

On arrival in our cardiac-surgical intensive care unit, the patient was in no distress. Mean blood pressure was 90 mm Hg by Doppler-modified manual sphygmomanometry. Jugular venous pressure was elevated, lungs were clear, and extremities were warm without edema. The EKG confirmed VF (Figure 1). Laboratory studies were unremarkable. The LVAD interrogation revealed slightly reduced flow.

**Figure 2.**
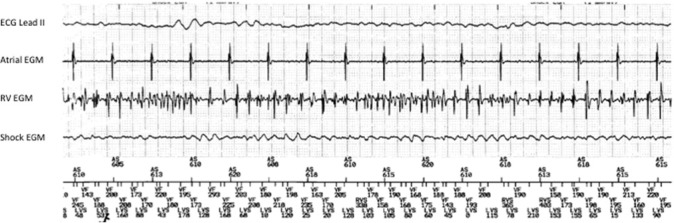
BiV-ICD interrogation demonstrating atrial systole in the atrial electrogram (Atrial EGM) and ventricular fibrillation in the ventricular electrogram (RV EGM). The surface ECG shows ventricular fibrillation (ECG Lead II).

A focused transthoracic echocardiogram (TTE) was performed to assess ventricular function. In the parasternal long-axis view (Clip 1; available online at http://hic.sagepub.com/content/by/supplemental-data), both the left and right ventricles are asystolic as expected with VF. Despite the complete lack of ventricular contraction, the mitral valve leaflets demonstrate normal mobility, and there is rhythmic contraction of the left atrial posterior wall. The aortic leaflets remain closed as expected in the presence of a functioning LVAD. Rhythmic contraction of the right atrium was visualized in the subcostal view (Clip 2; available online at http://hic.sagepub.com/content/by/supplemental-data), and the inferior vena cava was upper normal in size (1.9 cm) without inspiratory collapse. Subsequent interrogation of the BiV-ICD confirmed that the patient’s atria were contracting in sinus rhythm (Figure 2) despite VF.

Because of the risk of prolonged VF to RV function, the patient was sedated and successfully externally defibrillated using 200 Joules of asynchronous direct current biphasic waveform. Mexilitine was added to his medical regimen, and his carvedilol dose was increased. BiV-ICD tachytherapies were optimized. He was ultimately discharged home in stable condition.

## Discussion

Ventricular arrhythmias are common in patients with continuous-flow LVADs.^1^ LVAD patients usually have implantable defibrillators, which improve survival in this population.^2^ However, successful defibrillation can be limited by clinical and electrophysiologic characteristics including etiology of cardiac disease, ischemia, medications, duration of arrhythmia, and defibrillator lead configuration.^3^

Although LVADs maintain forward flow during arrhythmias, device preload can be impaired by the rhythm’s effect on RV function. Progressive RV dysfunction can result in decompensated heart failure and subsequent morbidity and mortality despite continued function of the LVAD.^4^

Two concerns in caring for an LVAD patient with refractory ventricular arrhythmias are therefore assessing adequacy of systemic flow and rapidly assessing the RV. Rapid assessment of LVAD function by LVAD interrogation and TTE is also important as inflow cannula malposition or inappropriate LVAD speed relative to a patient’s preload or afterload can precipitate ventricular arrhythmias.

This echocardiogram demonstrates a fascinating physiologic event, the simultaneous presence of atrial sinus rhythm and VF. The clinical scenario highlights the complexity of the care of continuous-flow LVAD patients and the challenges of intensivist-performed bedside focused TTE.

This study is available because it was performed at the bedside by an attending anesthesiologist/intensivist who was trained in focused echocardiography as part of a structured, proctored multidisciplinary program developed in conjunction with the Division of Cardiovascular Medicine. A component of this structured exam is limiting the scope to hemodynamically relevant points, which includes assessment of biventricular function, the primary reason for obtaining the study in this patient.

The presence of a continuous-flow LVAD added additional challenges for bedside echocardiography including the need for assessment of LVAD function.^5^ In this case, the patient’s heart failure cardiologist was present at the bedside during management, and reviewed TTE images in real time. This highlights another key component of critical-care echocardiography, multidisciplinary participation.

Abbreviated training in TTE for intensivists and other noncardiologists is evidence-supported and guidelines are being actively pursued by subspecialty societies.^6,7^ As with the development of intraoperative transesophageal echocardiography, guidelines, training, and ultimately certification will be critical given the potential dangers posed by inexperienced examiners.

In summary, we present a case of atrial sinus rhythm with simultaneous VF, which was diagnosed with TTE and confirmed with intracardiac electrograms obtained from BiV-ICD interrogation. The echocardiographic and physiologic findings are correlated, and the value of multidisciplinary, structured training in critical care echocardiography discussed.

## References

[1] Andersen M Videbaek R Boesgaard S Sander K Hansen PB Gustafsson F Incidence of ventricular arrhythmias in patients on long-term support with a continuous-flow assist device (HeartMate II). J Heart Lung Transplant. 2009;28:733-735. 19560703 10.1016/j.healun.2009.03.011

[2] Cantillon DJ Tarakji KG Kumbhani DJ Smedira NG Starling RC Wilkoff BL Improved survival among ventricular assist device recipients with concomitant implantable cardioverter-defibrillator. Heart Rhythm. 2010;4:466-471. 20178869 10.1016/j.hrthm.2009.12.022

[3] Shukia HH Flaker GC Jayam V Roberts D High defibrillation thresholds in transvenous biphasic implantable defibrillators: clinical predictors and prognostic implications. Pacing Clin Electrophysiol. 2003;26:44-48. 12685138 10.1046/j.1460-9592.2003.00148.x

[4] Baumwol J Macdonald PS Keogh AM Right heart failure and “failure to thrive” after left ventricular assist device: clinical predictors and outcomes. J Heart Lung Transplant. 2011;30:888-895. 21530314 10.1016/j.healun.2011.03.006

[5] Rasalingam R Johnson SN Bilhorn KR Transthoracic echocardiographic assessment of continuous flow left ventricular assist devices. J Am Soc Echocardiogr. 2011;24:135-148. 21236640 10.1016/j.echo.2010.11.012

[6] Faris JG Veltman MG Royse CF Limited transthoracic echocardiography assessment in anaesthesia and critical care. Best Pract Res Clin Anaesthesiol. 2009;23:285-298. 19862888 10.1016/j.bpa.2009.02.008

[7] Cowie BS Focused transthoracic echocardiography in the perioperative period. Anaesth Intensive Care. 2010;38:823-836. 20865866 10.1177/0310057X1003800505

